# Prevention and Control of Influenza with Vaccines: Recommendations of the Advisory Committee on Immunization Practices, United States, 2015–16 Influenza Season

**DOI:** 10.15585/mmwr.mm6430a3

**Published:** 2015-08-07

**Authors:** Lisa A. Grohskopf, Leslie Z. Sokolow, Sonja J. Olsen, Joseph S. Bresee, Karen R. Broder, Ruth A. Karron

**Affiliations:** 1 Influenza Division, National Center for Immunization and Respiratory Diseases, CDC; 2 Battelle Memorial Institute, Atlanta, Georgia; 3 Immunization Safety Office, National Center for Emerging and Zoonotic Infectious Diseases, CDC; 4 Johns Hopkins University, Baltimore, Maryland

This report updates the 2014 recommendations of the Advisory Committee on Immunization Practices (ACIP) regarding the use of seasonal influenza vaccines (1). Updated information for the 2015–16 season includes 1) antigenic composition of U.S. seasonal influenza vaccines; 2) information on influenza vaccine products expected to be available for the 2015–16 season; 3) an updated algorithm for determining the appropriate number of doses for children aged 6 months through 8 years; and 4) recommendations for the use of live attenuated influenza vaccine (LAIV) and inactivated influenza vaccine (IIV) when either is available, including removal of the 2014–15 preferential recommendation for LAIV for healthy children aged 2 through 8 years. Information regarding topics related to influenza vaccination that are not addressed in this report is available in the 2013 ACIP seasonal influenza recommendations (2).

Information in this report reflects discussions during public meetings of ACIP held on February 26 and June 24, 2015. Subsequent modifications were made during CDC clearance review to update information and clarify wording. Meeting minutes, information on ACIP membership, and information on conflicts of interest are available at http://www.cdc.gov/vaccines/acip/committee/members.html. Any updates will be posted at http://www.cdc.gov/flu.

## Groups Recommended for Vaccination and Timing of Vaccination

Routine annual influenza vaccination is recommended for all persons aged ≥6 months who do not have contraindications. Optimally, vaccination should occur before onset of influenza activity in the community. Health care providers should offer vaccination by October, if possible. Vaccination should continue to be offered as long as influenza viruses are circulating. Children aged 6 months through 8 years who require 2 doses (see “Vaccine Dose Considerations for Children Aged 6 Months through 8 Years”) should receive their first dose as soon as possible after vaccine becomes available, and the second dose ≥4 weeks later. To avoid missed opportunities for vaccination, providers should offer vaccination to unvaccinated persons aged ≥6 months during routine health care visits and hospitalizations when vaccine is available.

Antibody levels induced by vaccine decline after vaccination (3–5). Although a 2008 literature review found no clear evidence of more rapid decline among older adults (6), a 2010 study noted a statistically significant decline in antibody titers 6 months after vaccination among persons aged ≥65 years (5). A case-control study conducted in Navarre, Spain, during the 2011–12 influenza season revealed a decline in vaccine effectiveness, primarily affecting persons aged ≥65 years (7). While delaying vaccination might permit greater immunity later in the season, deferral might result in missed opportunities to vaccinate, as well as difficulties in vaccinating a population within a more constrained time period. Vaccination programs should balance maximizing the likelihood of persistence of vaccine-induced protection through the season with avoiding missed opportunities to vaccinate or vaccinating after influenza virus circulation begins.

## Influenza Vaccine Composition for the 2015–16 Season

For 2015–16, U.S.-licensed trivalent influenza vaccines will contain hemagglutinin (HA) derived from an A/California/7/2009 (H1N1)-like virus, an A/Switzerland/9715293/2013 (H3N2)-like virus, and a B/Phuket/3073/2013-like (Yamagata lineage) virus. This represents changes in the influenza A (H3N2) virus and the influenza B virus as compared with the 2014–15 season. Quadrivalent influenza vaccines will contain these vaccine viruses, and a B/Brisbane/60/2008-like (Victoria lineage) virus, which is the same Victoria lineage virus recommended for quadrivalent formulations in 2013–14 and 2014–15 (8).

## Available Vaccine Products and Indications

Various influenza vaccine products are anticipated to be available during the 2015–16 season (Table). These recommendations apply to all licensed influenza vaccines used within Food and Drug Administration (FDA)-licensed indications. Differences between ACIP recommendations and labeled indications are noted in the Table. For persons for whom more than one type of vaccine is appropriate and available, ACIP does not express a preference for use of any particular product over another.

New and updated influenza vaccine product approvals include the following:

1. In August 2014, FDA approved Afluria (inactivated influenza vaccine, bioCSL, Inc., King of Prussia, Pennsylvania) for intramuscular administration via the Stratis needle-free jet injector (PharmaJet, Inc., Golden, Colorado), for persons aged 18 through 64 years (9). Adults aged 18 through 64 years may receive Afluria either by the Stratis injector or with a sterile needle and syringe. All other inactivated influenza vaccines are approved for administration by sterile needle and syringe only. The Stratis injector is a reusable spring-powered device which injects the vaccine through a single-use sterile needle-free syringe into the deltoid muscle. In a prelicensure study of 1,250 adults aged 18 through 64 years (10), local injection site symptoms were reported more frequently by persons who received Afluria via the Stratis Injector than those who were vaccinated with a sterile needle and syringe; most resolved within 3 days. Those who received Afluria via the Stratis injector had antibody levels against influenza virus that were noninferior to those who received Afluria by sterile needle and syringe. Data comparing rates of influenza illness in persons vaccinated with the Stratis injector versus needle and syringe are not available.
2. In October 2014, FDA approved an expanded age indication for the use of Flublok (Recombinant Influenza Vaccine, Trivalent [RIV3], Protein Sciences, Meriden, Connecticut), which was previously approved for persons aged 18 through 49 years. Flublok is now indicated for persons aged ≥18 years (11). Approval for persons aged ≥50 years is based upon studies of immunogenicity and safety of the vaccine in three randomized trials (12–14); data demonstrating a decrease in influenza disease in persons aged ≥50 years after vaccination with Flublok are not available.
3. In December 2014, FDA approved Fluzone Intradermal Quadrivalent (Sanofi Pasteur, Inc., Swiftwater, Pennsylvania), for persons aged 18 through 64 years (15). It is anticipated that this formulation will replace the previously available trivalent Fluzone Intradermal for the 2015–16 influenza season. In a randomized study of 3,355 adults aged 18 through 64 years comparing safety and immunogenicity of Fluzone Intradermal Quadrivalent with two different trivalent intradermal formulations of Fluzone (each one containing one of the two influenza B viruses contained in the quadrivalent vaccine), the quadrivalent formulation was immunogenically noninferior to the trivalent formulations for the influenza A and matched B viruses, immunogenically superior for the unmatched B viruses, and had a similar adverse event profile (16). Efficacy data for Fluzone Intradermal Quadrivalent are not available.

## Vaccine Dose Considerations for Children Aged 6 Months Through 8 Years

Children aged 6 months through 8 years require 2 doses of influenza vaccine (administered ≥4 weeks apart) during their first season of vaccination to optimize response (17–19). Since the emergence of influenza A(H1N1)pdm09 (the 2009 H1N1 pandemic virus), recommendations for determining the number of doses needed have specified previous receipt of vaccine containing influenza A(H1N1)pdm09. In light of the continuing circulation of influenza A(H1N1)pdm09 as the predominant influenza A(H1N1) virus since 2009, and the inclusion of an A/California/7/2009(H1N1)-like virus in U.S. seasonal influenza vaccines since the 2010–2011 season, separate consideration of receipt of vaccine doses containing this virus is no longer recommended.

Several studies have suggested that for viruses which are the same in both doses of vaccine, longer intervals between the 2 doses do not compromise immune response (20–22). In a study conducted across two seasons during which the influenza A(H1N1) vaccine virus did not change but the B virus did change, children aged 10 through 24 months who received 1 dose of IIV during the fall of each season had similar immune responses to the unchanged A(H1N1) virus antigen and to the drifted A(H3N2) virus antigen, compared with children aged 6 through 24 months who received 2 doses of the same IIV during the latter season. However, the first group had significantly lower antibody responses to the B antigen (20). Since the 2010–11 season, guidance for determining the appropriate number of doses has taken strain changes into account. Because of the change in vaccine composition for 2015–16, children aged 6 months through 8 years will need to have received ≥2 doses of influenza vaccine previously to require only 1 dose for the 2015–16 season.

**FIGURE 1:**
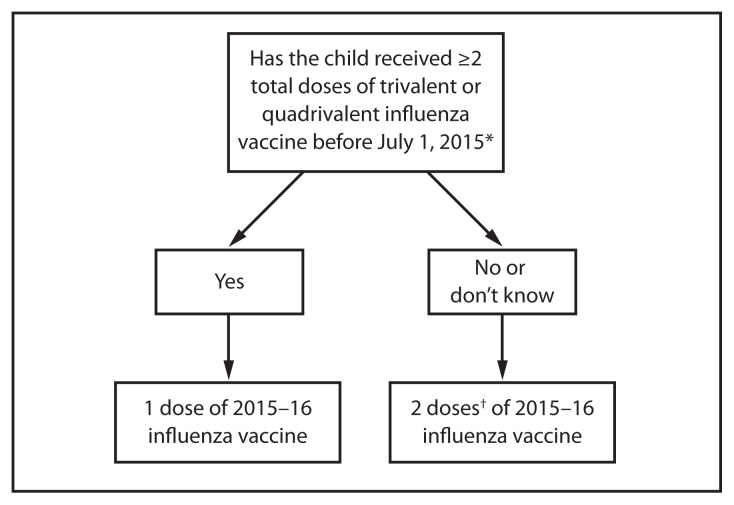
Influenza vaccine dosing algorithm for children aged 6 months through 8 years — Advisory Committee on Immunization Practices, United States, 2015–16 influenza season *The two doses need not have been received during the same season or consecutive seasons. ^†^Doses should be administered ≥4 weeks apart.

For 2015–16, ACIP recommends that children aged 6 months through 8 years who have previously received ≥2 total doses of trivalent or quadrivalent influenza vaccine before July 1, 2015, require only 1 dose for 2015–16. The two previous doses need not have been given during the same season or consecutive seasons. Children in this age group who have not previously received a total of ≥2 doses of trivalent or quadrivalent influenza vaccine before July 1, 2015 require 2 doses for 2015–16. The interval between the 2 doses should be at least 4 weeks (Figure 1).

## Considerations for the Use of Live Attenuated Influenza Vaccine and Inactivated Influenza Vaccine When Either is Available

Both LAIV and IIV have been demonstrated to be effective in children and adults. Among adults, most comparative studies have demonstrated that LAIV and IIV were of similar efficacy or that IIV was more efficacious (23). Several studies conducted before the 2009 H1N1 pandemic demonstrated superior efficacy of LAIV in children (24–26). A randomized controlled trial conducted during the 2004–05 season among 7,852 children aged 6 through 59 months demonstrated a 55% reduction in culture-confirmed influenza among children who received trivalent LAIV (LAIV3) compared with those who received trivalent IIV (IIV3). LAIV3 efficacy was higher than that of IIV3 against both antigenically drifted and well-matched influenza viruses (24). In a comparative study conducted during the 2002–03 season, LAIV3 provided 53% greater relative efficacy compared with IIV3 in children aged 6 through 71 months who had previously experienced recurrent respiratory tract infections (25).

In June 2014, following review of evidence on the relative efficacy of LAIV compared with IIV for healthy children, ACIP recommended that when immediately available, LAIV should be used for healthy children aged 2 through 8 years who have no contraindications or precautions. However, data from subsequent observational studies of LAIV and IIV vaccine effectiveness indicated that LAIV did not perform as well as expected based upon the observations in earlier randomized trials (27,28). Analysis of data from three observational studies of LAIV4 vaccine effectiveness for the 2013–14 season (the first season in which LAIV4 was available) revealed poor effectiveness of LAIV4 against influenza A(H1N1)pdm09 among children aged 2 through 17 years (27). During this season, H1N1pdm09 virus predominated for the first time since the 2009 pandemic. The reasons for the lack of effectiveness of LAIV4 against influenza A(H1N1)pdm09 are still under investigation. Moreover, although one large randomized trial observed superior relative efficacy of LAIV3 compared with IIV3 against antigenically drifted H3N2 influenza viruses during the 2004–05 season (24), interim analysis of observational data from the U.S. Influenza Vaccine Effectiveness (U.S. Flu VE) Network for the early 2014–15 season (in which antigenically drifted H3N2 viruses were predominant) indicated that neither LAIV4 nor IIV provided significant protection in children aged 2 through 17 years; LAIV did not offer greater protection than IIV for these viruses (28).

In the absence of data demonstrating consistent greater relative effectiveness of the current quadrivalent formulation of LAIV, preference for LAIV over IIV is no longer recommended. ACIP will continue to review the effectiveness of influenza vaccines in future seasons and update these recommendations if warranted.

For children and adults with chronic medical conditions conferring a higher risk for influenza complications, data on the relative safety and efficacy of LAIV and IIV are limited. In a study comparing LAIV3 and IIV3 among children aged 6 through 17 years with asthma conducted during the 2002–03 season, LAIV conferred 32% increased protection relative to IIV in preventing culture-confirmed influenza; no significant difference in asthma exacerbation events was noted (26). Available data are insufficient to determine the level of severity of asthma for which administration of LAIV would be appropriate.

For 2015–16, ACIP recommends the following:

1. All persons aged ≥6 months should receive influenza vaccine annually. Influenza vaccination should not be delayed to procure a specific vaccine preparation if an appropriate one is already available.
2. For healthy children aged 2 through 8 years who have no contraindications or precautions, either LAIV or IIV is an appropriate option. No preference is expressed for LAIV or IIV for any person aged 2 through 49 years for whom either vaccine is appropriate. An age-appropriate formulation of vaccine should be used.
3. LAIV should not be used in the following populations:
  – Persons aged <2 years or >49 years;
  – Persons with contraindications listed in the package insert:
    ○ Children aged 2 through 17 years who are receiving aspirin or aspirin-containing products;
    ○ Persons who have experienced severe allergic reactions to the vaccine or any of its components, or to a previous dose of any influenza vaccine;
  – Pregnant women;
  – Immunocompromised persons (see also “Vaccine Selection and Timing of Vaccination for Immunocompromised Persons”);
  – Persons with a history of egg allergy;
  – Children aged 2 through 4 years who have asthma or who have had a wheezing episode noted in the medical record within the past 12 months, or for whom parents report that a health care provider stated that they had wheezing or asthma within the last 12 months (Table, footnote). For persons aged ≥5 years with asthma, recommendations are described in item 4 of this list;
  – Persons who have taken influenza antiviral medications within the previous 48 hours.
4. In addition to the groups for whom LAIV is not recommended above, the “Warnings and Precautions” section of the LAIV package insert indicates that persons of any age with asthma might be at increased risk for wheezing after administration of LAIV (29). The package insert also notes that the safety of LAIV in persons with other underlying medical conditions that might predispose them to complications after wild-type influenza virus infection (e.g., chronic pulmonary, cardiovascular [except isolated hypertension], renal, hepatic, neurologic, hematologic, or metabolic disorders [including diabetes mellitus]) (2), has not been established. These conditions, in addition to asthma in persons aged ≥5 years, should be considered precautions for the use of LAIV.
5. Persons who care for severely immunosuppressed persons who require a protective environment should not receive LAIV, or should avoid contact with such persons for 7 days after receipt, given the theoretical risk for transmission of the live attenuated vaccine virus to close contacts.

## Influenza Vaccination of Persons With a History of Egg Allergy

Severe allergic and anaphylactic reactions can occur in response to various influenza vaccine components, but such reactions are rare. With the exceptions of recombinant influenza vaccine (RIV3, Flublok) and cell-culture based inactivated influenza vaccine (ccIIV3, Flucelvax, Novartis, Cambridge, Massachusetts), currently available influenza vaccines are prepared by propagation of virus in embryonated eggs. A 2012 review of published data, including 4,172 egg-allergic patients (513 reporting a history of severe allergic reaction) noted no occurrences of anaphylaxis following administration of IIV3, though some milder reactions did occur (30). This suggests that severe allergic reactions to egg-based influenza vaccines are unlikely. On this basis, some guidance recommends that no additional measures are needed when administering influenza vaccine to egg-allergic persons (31). However, occasional cases of anaphylaxis in egg-allergic persons have been reported to the Vaccine Adverse Event Reporting System (VAERS) after administration of influenza vaccine (32,33). IIVs containing as much as 0.7 *μ*g/0.5 mL have reportedly been tolerated (34,35); however, a threshold below which no reactions would be expected is not known (34). Among IIVs for which ovalbumin content was disclosed during the 2011–12 through 2014–15 seasons, reported maximum amounts were ≤1 *μ*g/0.5 mL dose; however, not all manufacturers disclose this information in the package inserts. Ovalbumin is not directly measured for Flucelvax, but it is estimated by calculation from the initial content in the reference virus strains to contain less than 5×10^−8^ *μ*g/0.5 mL dose of total egg protein, of which ovalbumin is a fraction (Novartis, unpublished data, 2013). Flublok is considered egg-free. However, neither Flucelvax nor Flublok is licensed for children aged <18 years.

Compared with IIV, fewer data are available concerning the use of LAIV in the setting of egg allergy. In a prospective cohort study of children aged 2 through 16 years (69 with egg allergy and 55 without), all of whom received LAIV, none of the egg-allergic subjects developed signs or symptoms of an allergic reaction during the one hour of postvaccination observation, and none reported adverse reactions that were suggestive of allergic reaction or which required medical attention after 24 hours (36). In a larger study of 282 egg-allergic children aged 2 through 17 years (115 of whom had experienced anaphylactic reactions to egg previously), no systemic allergic reactions were observed after LAIV administration. On the basis of these data, the upper limit of the 95% confidence interval for the incidence of a systemic allergic reaction (including anaphylaxis) in children with egg allergy was estimated to be 1.3% (37). Eight children experienced milder, self-limited symptoms which may have been caused by an IgE-mediated reaction. ACIP will continue to review safety data for use of LAIV in the setting of egg allergy.

**FIGURE 2:**
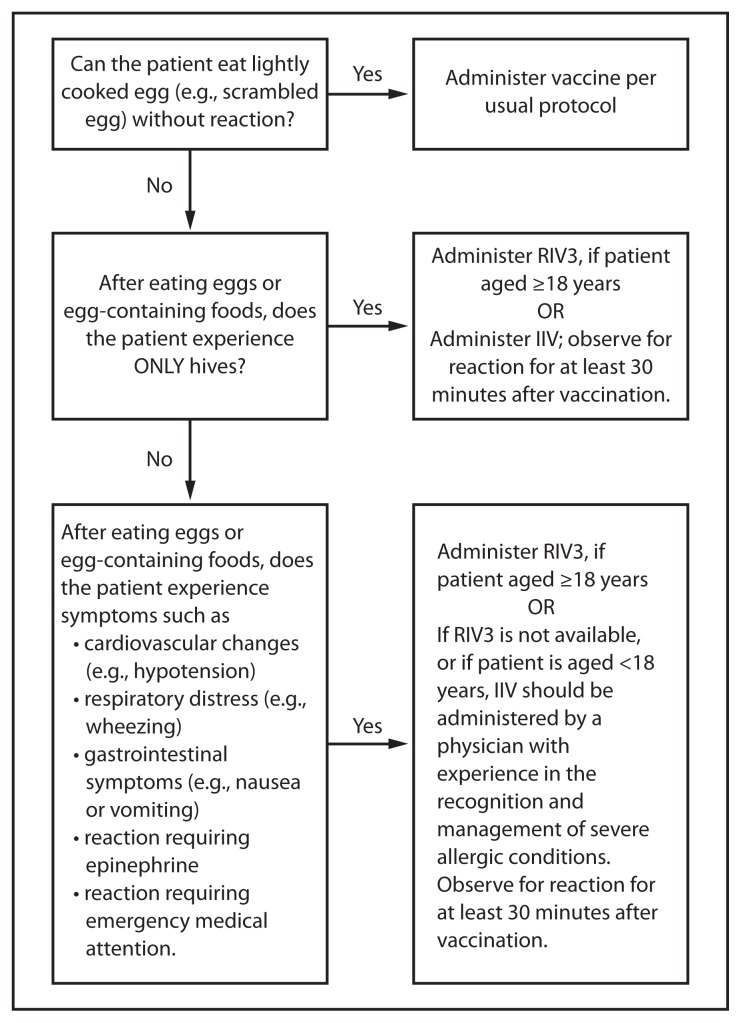
Recommendations regarding influenza vaccination of persons who report allergy to eggs*^†^ — Advisory Committee on Immunization Practices, United States, 2015–16 influenza season **Abbreviations:** IIV = inactivated influenza vaccine, trivalent or quadrivalent; RIV3 = recombinant influenza vaccine, trivalent. * Persons with egg allergy may tolerate egg in baked products (e.g., bread or cake). Tolerance to egg-containing foods does not exclude the possibility of egg allergy (Erlewyn-Lajeunesse et al., Recommendations for the administration of influenza vaccine in children allergic to egg. BMJ 2009;339:b3680). ^†^ For persons who have no known history of exposure to egg, but who are suspected of being egg-allergic on the basis of previously performed allergy testing, consultation with a physician with expertise in the management of allergic conditions should be obtained prior to vaccination. Alternatively, RIV3 may be administered if the recipient is aged ≥18 years.

For the 2015–16 influenza season, ACIP recommends the following:

1. Persons with a history of egg allergy who have experienced only hives after exposure to egg should receive influenza vaccine. Because relatively few data are available for use of LAIV in this setting, IIV or trivalent recombinant influenza vaccine (RIV3) should be used. RIV3 may be used for persons aged ≥18 years who have no other contraindications. However, IIV (egg- or cell culture-based) may also be used, with the following additional safety measures (Figure 2):
  – Vaccine should be administered by a health care provider who is familiar with the potential manifestations of egg allergy; and
  – Vaccine recipients should be observed for ≥30 minutes for signs of a reaction after administration of each vaccine dose.
2. Persons who report having had reactions to egg involving such symptoms as angioedema, respiratory distress, lightheadedness, or recurrent emesis; or who required epinephrine or another emergency medical intervention, may receive RIV3 if they are aged ≥18 years and there are no other contraindications. If RIV3 is not available or the recipient is not within the indicated age range, IIV should be administered by a physician with experience in the recognition and management of severe allergic conditions (Figure 2).
3. Regardless of allergy history, all vaccines should be administered in settings in which personnel and equipment for rapid recognition and treatment of anaphylaxis are available (38).
4. Persons who are able to eat lightly cooked egg (e.g., scrambled egg) without reaction are unlikely to be allergic. Egg-allergic persons might tolerate egg in baked products (e.g., bread or cake). Tolerance to egg-containing foods does not exclude the possibility of egg allergy. Egg allergy can be confirmed by a consistent medical history of adverse reactions to eggs and egg-containing foods, plus skin and/or blood testing for immunoglobulin E directed against egg proteins (39).
5. For persons with no known history of exposure to egg, but who are suspected of being egg-allergic on the basis of previously performed allergy testing, consultation with a physician with expertise in the management of allergic conditions should be obtained before vaccination (Figure 2). Alternatively, RIV3 may be administered if the recipient is aged ≥18 years.
6. A previous severe allergic reaction to influenza vaccine, regardless of the component suspected of being responsible for the reaction, is a contraindication to future receipt of the vaccine.

## Vaccine Selection and Timing of Vaccination for Immunocompromised Persons

Immunocompromised states are caused by a heterogeneous range of conditions. In many instances, limited data are available regarding the use of influenza vaccines in the setting of specific immunocompromised states. In general, live virus vaccines, such as LAIV, should not be used for persons with most forms of altered immunocompetence (38). The Infectious Diseases Society of America (IDSA) has published detailed guidance for the selection and timing of vaccines for persons with specific immunocompromising conditions, including congenital immune disorders, stem cell and solid organ transplant, anatomic and functional asplenia, and therapeutic drug-induced immunosuppression, as well as for persons with cochlear implants or other conditions leading to persistent cerebrospinal fluid-oropharyngeal communication (40). ACIP will continue to review accumulating data on use of influenza vaccines in these contexts.

## Figures and Tables

**TABLE:** Influenza vaccines — United States, 2015–16 influenza season*

Trade name	Manufacturer	Presentation	Mercury (from thimerosal) *μ*g/0.5 mL	Ovalbumin *μ*g/0.5 mL	Age indications	Latex	Route
**Inactivated influenza vaccine, quadrivalent (IIV4), standard dose**							
*Contraindications* * *: Severe allergic reaction to any vaccine component, including egg protein, or after previous dose of any influenza vaccine.*							
*Precautions* * *: Moderate to severe acute illness with or without fever; history of Guillain-Barré syndrome within 6 weeks of receipt of influenza vaccine.*							
Fluarix Quadrivalent	GlaxoSmithKline	0.5 mL single-dose prefilled syringe	—	≤0.05	≥3 yrs	No	IM†
FluLaval Quadrivalent	ID Biomedical Corp. of Quebec (distributed by GlaxoSmithKline)	5.0 mL multi-dose vial	<25	≤0.3	≥3 yrs	No	IM†
Fluzone Quadrivalent	Sanofi Pasteur	0.25 mL single-dose prefilled syringe	—	§	6 through 35 mos	No	IM†
		0.5 mL single-dose prefilled syringe	—	§	≥36 mos	No	IM†
		0.5 mL single-dose vial	—	§	≥36 mos	No	IM†
		5.0 mL multi-dose vial	25	§	≥6 mos	No	IM†
Fluzone Intradermal¶ Quadrivalent	Sanofi Pasteur	0.1 mL single-dose prefilled microinjection system	—	§	18 through 64 yrs	No	ID**
**Inactivated influenza vaccine, trivalent (IIV3), standard dose**							
*Contraindications* * *: Severe allergic reaction to any vaccine component, including egg protein, or after previous dose of any influenza vaccine.*							
*Precautions* * *: Moderate to severe acute illness with or without fever; history of Guillain-Barré syndrome within 6 weeks of receipt of influenza vaccine.*							
Afluria	bioCSL	0.5 mL single-dose prefilled syringe	—	<1	≥9 yrs††	No	IM†
		5.0 mL multi-dose vial	24.5	<1	≥9 yrs†† via needle;18 through 64 yrs via jet injector	No	IM†
Fluvirin	Novartis Vaccines and Diagnostics	0.5 mL single-dose prefilled syringe	≤1	≤1	≥4 yrs	Yes§§	IM†
		5.0 mL multi-dose vial	25	≤1	≥4 yrs	No	IM†
Fluzone	Sanofi Pasteur	5.0 mL multi-dose vial	25	§	≥6 mos	No	IM†
**Inactivated influenza vaccine, cell-culture-based (ccIIV3), standard dose**							
*Contraindications* * *: Severe allergic reaction to any vaccine component, including egg protein, or after previous dose of any influenza vaccine.*							
*Precautions* * *: Moderate to severe acute illness with or without fever; history of Guillain-Barré syndrome within 6 weeks of receipt of influenza vaccine.*							
Flucelvax	Novartis Vaccines and Diagnostics	0.5 mL single-dose prefilled syringe	—	¶¶	≥18 yrs	Yes§§	IM†
**Inactivated influenza vaccine, trivalent (IIV3), high dose**							
*Contraindications* * *: Severe allergic reaction to any vaccine component, including egg protein, or after previous dose of any influenza vaccine.*							
*Precautions* * *: Moderate to severe acute illness with or without fever; history of Guillain-Barré syndrome within 6 weeks of receipt of influenza vaccine.*							
Fluzone High-Dose***	Sanofi Pasteur	0.5 mL single-dose prefilled syringe	—	§	≥65 yrs	No	IM†
**Recombinant influenza vaccine, trivalent (RIV3), standard dose**							
*Contraindications* * *: Severe allergic reaction to any vaccine component.*							
*Precautions* * *: Moderate to severe acute illness with or without fever; history of Guillain-Barré syndrome within 6 weeks of receipt of influenza vaccine.*							
Flublok	Protein Sciences	0.5 mL single-dose vial	—	0	≥18 yrs	No	IM†
**Live attenuated influenza vaccine, quadrivalent (LAIV4)**							
*Contraindications* * *: Severe allergic reaction to any vaccine component, including egg protein, or after previous dose of any influenza vaccine. Concomitant use of aspirin or aspirin-containing medications in children and adolescents.*							
*In addition, ACIP recommends LAIV4 not be used for pregnant women, immunosuppressed persons, persons with egg allergy, and children aged 2 through 4 years who have asthma or who have had a wheezing episode noted in the medical record within the past 12 months, or for whom parents report that a health care provider stated that they had wheezing or asthma within the last 12 months.*							
*LAIV4 should not be administered to persons who have taken influenza antiviral medications within the previous 48 hours.*							
*Persons who care for severely immunosuppressed persons who require a protective environment should not receive LAIV4, or should avoid contact with such persons for 7 days after receipt.*							
*Precautions* * *: Moderate to severe acute illness with or without fever; history of Guillain-Barré syndrome within 6 weeks of receipt of influenza vaccine; asthma in persons aged 5 years and older; medical conditions which might predispose to higher risk for complications attributable to influenza.*							
FluMist Quadrivalent†††	MedImmune	0.2 mL single-dose prefilled intranasal sprayer	—	<0.24 (per 0.2 mL)	2 through 49 yrs	No	IN

**Abbreviations:** ACIP = Advisory Committee on Immunization Practices; ID = intradermal; IM = intramuscular; IN = intranasal.

* Immunization providers should check Food and Drug Administration-approved prescribing information for 2015–16 influenza vaccines for the most complete and updated information, including (but not limited to) indications, contraindications, warnings, and precautions. Package inserts for U.S.-licensed vaccines are available at www.fda.gov/BiologicsBloodVaccines/Vaccines/ApprovedProducts/ucm093833.htm.

† For adults and older children, the recommended site for intramuscular influenza vaccination is the deltoid muscle. The preferred site for infants and young children is the anterolateral aspect of the thigh. Specific guidance regarding site and needle length for intramuscular administration may be found in the ACIP General Recommendations on Immunization, available at www.cdc.gov/mmwr/preview/mmwrhtml/rr6002a1.htm.

§ Available upon request from Sanofi Pasteur (1–800–822–2463 or MIS.Emails@sanofipasteur.com).

¶ Quadrivalent inactivated influenza vaccine, intradermal: a 0.1-mL dose contains 9 *μ*g of each vaccine antigen (36 *μ*g total).

** The preferred injection site is over the deltoid muscle. Fluzone Intradermal Quadrivalent is administered using the delivery system included with the vaccine.

†† Age indication per package insert is ≥5 years; however, ACIP recommends Afluria not be used in children aged 6 months through 8 years because of increased risk of febrile reactions noted in this age group with bioCSL’s 2010 Southern Hemisphere IIV3. If no other age-appropriate, licensed inactivated seasonal influenza vaccine is available for a child aged 5 through 8 years who has a medical condition that increases the child’s risk for influenza complications, Afluria can be used; however, providers should discuss with the parents or caregivers the benefits and risks of influenza vaccination with Afluria before administering this vaccine. Afluria may be used in persons aged ≥9 years.

§§ Syringe tip cap may contain natural rubber latex.

¶¶ Information not included in package insert. Estimated to contain <50 femtograms (5×10^−8^ *μ*g) of total egg protein (of which ovalbumin is a fraction) per 0.5 mL dose of Flucelvax.

*** Trivalent inactivated influenza vaccine, high-dose: a 0.5-mL dose contains 60 *μ*g of each vaccine antigen (180 *μ*g total).

††† FluMist is shipped refrigerated and stored in the refrigerator at 35°F–46°F (2°C–8°C) after arrival in the vaccination clinic. The dose is 0.2 mL divided equally between each nostril. Health care providers should consult the medical record, when available, to identify children aged 2 through 4 years with asthma or recurrent wheezing that might indicate asthma. In addition, to identify children who might be at greater risk for asthma and possibly at increased risk for wheezing after receiving LAIV, parents or caregivers of children aged 2 through 4 years should be asked: “In the past 12 months, has a health care provider ever told you that your child had wheezing or asthma?” Children whose parents or caregivers answer “yes” to this question and children who have asthma or who had a wheezing episode noted in the medical record within the past 12 months should not receive FluMist.
